# A Liberal Doctor

**Published:** 1884-05-20

**Authors:** 


### A LIBERAL DOCTOR.

Dr. J. M. Toner, of Washington City, has donated to the Government Library, 27,515 volumes, and 15,755 pamphlets; being the life time accumulations of his private library in which he had taken great interest—being a man especially devoted to books.

